# 454-Pyrosequencing: A Molecular Battiscope for Freshwater Viral Ecology

**DOI:** 10.3390/genes1020210

**Published:** 2010-07-21

**Authors:** David J. Rooks, Darren L. Smith, James E. McDonald, Martin J. Woodward, Alan J. McCarthy, Heather E. Allison

**Affiliations:** 1 Microbiology Research Group, School of Biological Sciences, Biosciences Building, University of Liverpool, Crown Street, Liverpool, L69 7ZB, UK; E-Mails: david.rooks@liv.ac.uk (D.J.R.); smithdx@liv.ac.uk (D.L.S.); j.mcdonald@bangor.ac.uk (J.E.M.); aj55m@liv.ac.uk (A.J.M.); 2 Veterinary Laboratories Agency (Weybridge), New Haw, Addlestone, Surrey KT15 3NB, UK; E-Mail: m.j.woodward@vla.defra.gsi.gov.uk; † Present address: Liverpool HIV Pharmacology Group, The University of Liverpool, Pembroke Place, Liverpool, L69 3GF, UK; ‡ Present address: School of Biological Sciences, Bangor University, Deiniol Road, Bangor, Gwynedd, LL57 2UW, UK

**Keywords:** virome production, viral pathogens, pathogen dissemination, metagenomics, viral ecology

## Abstract

Viruses, the most abundant biological entities on the planet, are capable of infecting organisms from all three branches of life, although the majority infect bacteria where the greatest degree of cellular diversity lies. However, the characterization and assessment of viral diversity in natural environments is only beginning to become a possibility. Through the development of a novel technique for the harvest of viral DNA and the application of 454 pyrosequencing, a snapshot of the diversity of the DNA viruses harvested from a standing pond on a cattle farm has been obtained. A high abundance of viral genotypes (785) were present within the virome. The absolute numbers of lambdoid and Shiga toxin (Stx) encoding phages detected suggested that the depth of sequencing had enabled recovery of only *ca.* 8% of the total virus population, numbers that agreed within less than an order of magnitude with predictions made by rarefaction analysis. The most abundant viral genotypes in the pond were bacteriophages (93.7%). The predominant viral genotypes infecting higher life forms found in association with the farm were pathogens that cause disease in cattle and humans, e.g. members of the *Herpesviridae.* The techniques and analysis described here provide a fresh approach to the monitoring of viral populations in the aquatic environment, with the potential to become integral to the development of risk analysis tools for monitoring the dissemination of viral agents of animal, plant and human diseases.

## 1. Introduction 

Viruses, most of which infect microorganisms, are the most abundant biological entities on the planet and are capable of infecting organisms from all three branches of life [1]. Because bacteria represent the greatest portion of microbial life and diversity of earth, bacteriophages form the largest division of the viruses and have the potential to alter the genetic composition, and ultimately the functioning of microbial communities [2]. Viruses are important, possibly the most important, drivers of microbial evolution; resistance to infection can be due to the alteration of surface epitopes or the development of alternative intracellular anti-viral mechanisms [3]. In their role as agents of genetic exchange, they not only alter the genetic makeup of host cells, but also play a pivotal role in the movement of genetic material between ecosystems [4].

However the characterization and assessment of viral diversity in the natural environment is hindered by the limitations of current isolation and recovery methods, and the specificity requirements of individual propagation and identification techniques. The size, nature, rapid evolution and genomic flexibility of viruses make them notoriously difficult to study [5,6]. Propagation of viruses is also likely to be untenable in many cases because their hosts cannot be isolated or cultured in the laboratory [7]. For example, the greatest diversity is found amongst the bacteriophages yet it has been predicted that less than 0.0002% of the global bacteriophage pangenome has been sampled thus far [8]. Studies of viral ecology are further constrained by the absence of single genetic elements that are shared by all viruses [9,10]. Although genome comparisons have shown that conserved genes are present within certain groups such as the lambdoid phages [11], these genes will not enable the identification of novel viral groups [12], and viral diversity cannot be determined by using approaches analogous to the cellular RNA profiling approach that has been so successful in revealing the untold diversity within Bacteria and Archaea [1].

Metagenomics, or community genomics, is an approach aimed at analyzing the genomic content of microbial communities within a particular niche [13]. Early metagenomic projects, for example those of Beja and coworkers [13,14,15], were plagued by potential biases imparted mainly by DNA extraction and cloning methods [16]. The traditional approach to metagenome sequencing involves cloning DNA into BACs, fosmids or cosmids and the use of dideoxy chain-termination sequencing [17]. This approach is not well suited to viral metagenomics because some viruses possess an RNA-based genome, DNA viruses often contain extremely small genomes [18] with modified bases making cloning difficult [19], and they can carry genes or sequences that are toxic to bacterial cloning hosts [20]. An alternative approach is the use of next generation sequencing techniques, which provide unbiased, high volumes of short DNA sequences without the requirement for cloning [21]. Pyrosequencing technology has already been used to analyze viral communities in a range of natural environments, including but not exclusive to the examination of the structural and functional changes in coral microbiota [22], the diversity, composition and adaptations of viral assemblages in hot springs [23], Antarctic lake viral communities [24], marine viral communities [5,25] and fresh water communities [26,27] all of which revealed unexpected genetic richness. Viral metagenomic studies have shown that 1 kg of marine sediment contains over a million different viral genotypes [28,29] and at least 1000 unique novel viruses can be identified in the human gut, though the vast majority (>70%) are completely uncharacterized [30]. Many microbial metagenomic projects based on pyrosequencing are limited by complex assembly issues associated with small sequence reads sampled from a mixed community of large genomes; these limitations are less significant in viral metagenomics, due to the relatively small size range of viral genomes [31]. In addition, the hardiness of the viral capsid can be exploited to concentrate and purify viruses away from contaminating microorganisms, prior to sequencing [32].

This study reports a snapshot examination of the diversity of the DNA viruses present in a standing pond on a cattle farm in Cheshire, UK, selected for study as a result of previous work on Shiga toxin (Stx) encoding bacteriophages in relation to the bovine reservoir of *E. coli* strains pathogenic to man [11,33,34,35]. This was achieved through the development of a novel technique for harvesting uncontaminated viral DNA [11], coupled with the application of 454 pyrosequencing to the viral metagenome so produced. Freshwater viral diversity and community structure has barely been studied, yet it is an important topic that impacts on human and animal disease with implications for environmental monitoring, agricultural practices and public health policy.

## 2. Results and Discussion

### 2.1. Preparation of DNA

A protocol was developed for the extraction of viral DNA from water samples, with the ultimate goal of minimising the presence of cellular and free DNA. In order to maximize the recovery of viral DNA, removal of bacterial and small eukaryote cells by membrane filtration was avoided as viruses can become immobilized on and trapped within filter membranes [5]. The efficacy of the DNA preparation method was evaluated both pre- and post-sequencing. Pre-sequencing, the samples were subjected to 35 cycles of DNA amplification by PCR using universal oligonucleotide primer sets for the 16S and 18S rRNA genes (Table 1). The inability to amplify bacterial or eukaryotic rRNA genes from the environmental viral DNA sample, as determined by ethidium bromide staining of DNA bands separated by agarose gel electrophoresis with all appropriate positive and negative controls (data not shown), demonstrated that cellular DNA contamination was at a level that could not be detected by PCR amplification and was therefore potentially suitable for use as the template for 454 pyrosequencing. 

**Table 1:** Oligonucleotide primers used in this study.

Gene target	Primername	Sequence (5’ -> 3’)	Annealing temperature (°C)	Amplicon size (bp)	Reference
16S rRNA	pA	AGAGTTTGATCCTGGCTCAG	55	1534	[36]
	pH	aaggaggtgatccsgccgca			
18S rRNA	NS1-Euk	ccagtagtcatatgcttgtc	50	1600	[37]
	Univ 1390	gacgggcggtgtgtacaa			

### 2.2. Metagenomic library output 

The 454 pyrosequencing returned 69,162 reads of DNA sequence, which contained 13,669,562 bp of DNA with a fragment size range of 32-404 bp using ¼ of a sequencing picotitre plate (85% of the sequences were >100 bp and > 51% of the sequences were between 200 – 250 bp, Supplementary Table 1). The DNA sequence reads were used directly for downstream analysis, and not assembled as contiguous sequences. The mosaic nature of viruses, particularly bacteriophage genomes [38], is such that subjecting the sequence data to conventional assembly of contigs would be inappropriate. Consequently, pre-packaged bioinformatic tools and computational approaches were applied to unravel the population structure and function of the viral metagenome sample. The methods used here for the prediction of identities from the metagenomic sequence data were largely automated, involving the use of MEGAN [39] and MG-RAST[40] coupled with BLAST [41] for the comparison of this viral metagenome against the non-redundant viral genome [42] and SEED databases (which uses a non-redundant database) [43]. 

### 2.3. Post-pyrosequencing analyses 

Using MG-RAST, sequences were screened for ribosomal RNA genes using BLASTn against the rRNA gene databases, which were extracted from Greengenes [44] the Ribosomal Database Project (RDP) [45] and the European Ribosomal Database Project (ERDP) [46] using cutoff parameters requiring similarity >5 bp in length and an expect value of less than 1 x 10^-5^[40]. Of the 69,162 sequences generated, 18,931 (27.37%) were classified (the SEED database assigns a metabolic potential to these sequences) and 50,231 (72.6%) were unclassified (the SEED database fails to assign a potential function to these sequences. Two of the 69,162 sequences possessed homology (Table 2) to SSU rRNA . The first SSU rRNA sequence (254 bp in length) was 97% identical to *Dechloromonas*, a member of the *betaproteobacteria* and further BLAST analysis of these sequences only identified organisms isolated from freshwater environments (e.g. accession numbers DQ450182 and DQ22839). The second SSU rRNA sequence (243 bp in length) possessed 95% identity to an unclassified member of the Bacteriodetes. It cannot be ruled out at this point that bacteriophages do not acquire portions or intact copies of SSU rRNA molecules [47], but additionally the low numbers of identified SSU rRNA sequences were below the level of detection by PCR amplification and indicate that the viral metagenomic library was at least heavily enriched for non-cellular DNA. 

### 2.4. Functional analyses of the virome

Gene transfer undoubtedly occurs in the natural environment, however the scale of the process and the implications for the evolution of the virus and its host organism is poorly understood [25]. Horizontal gene transfer of biochemically important genes from host to virus, virus to host and so on, will ultimately shape the microbial biosphere. 

**Table 2:** MG-RAST sequence classification.

	SSU rRNA homology (%) RDP/ERDP/GREENGENES	Protein Based (%)(SEED database*)
**Archaea**	0	1.99
**Bacteria**	0.02	41.71
**Eukaryota**	0	3.10
**Virus**	0	51.25
**Other**	0	0.02

*Used to determine the metabolic profile of the metagenome.

**Figure 1:**
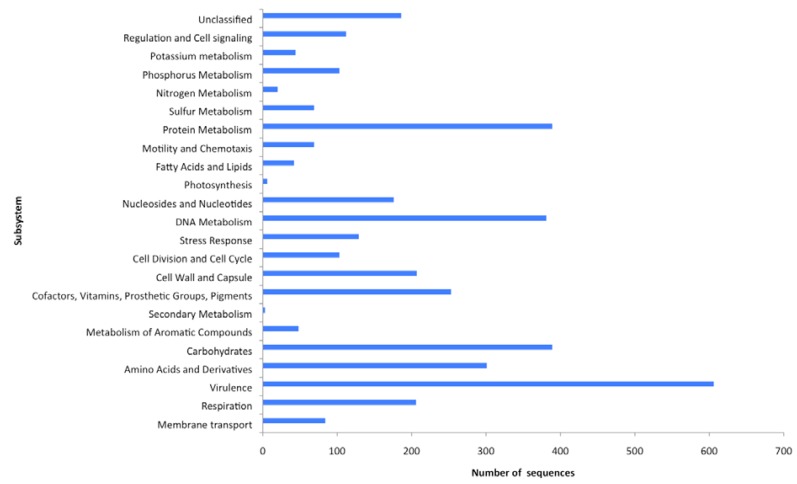
Functional potential of sequences from the freshwater virome. This was calculated using a BLASTX algorithm against the SEED database. An E-value of <0.001 was considered to be specific.

**Figure 2:**
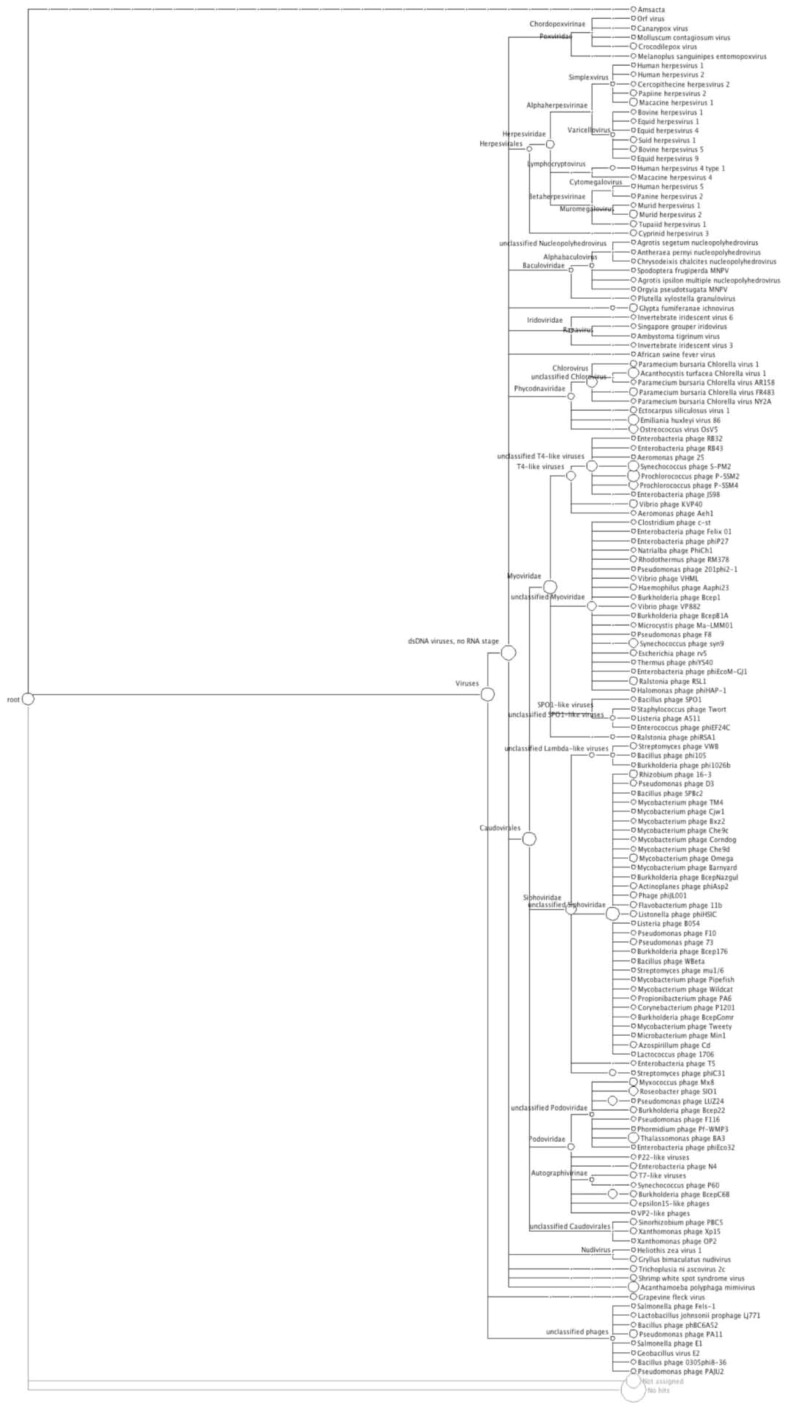
Phylogenetic diversity of the freshwater metagenome as calculated by MEGAN. Each circle represents a taxon in the NCBI taxonomy, and the size of the circle is scaled logarithmically to represent the number of reads assigned directly to the taxon. This Figure is also available as supplementary file.

Metagenomics allows the analysis of the complete genetic information contained within the viral community, and in this way the relative abundances of all genes can be determined and used to generate a description of the functional potential of each viral community within a given sample [5,29,30,48,49,50,51]. The metabolic potential of the virome was determined using the SEED database (Table 2, Supplementary Table 2) [43], which comprises all known protein sequences spanning all biological taxonomies [43]. The SEED arranges metabolic pathways into a hierarchical structure in which all of the genes required for a specific task are arranged into subsystems. At the highest level of organization, the subsystems include both catabolic and anabolic functions, for example DNA metabolism, and at the lowest level the subsystems are specific pathways, for example the synthesis pathway for thymidine [26]. It uses a BLASTX algorithm and assigns genes to subsystems, each of which is composed of a group of functionally related proteins where an E-value of <0.001 is considered to be significant [43] so that the genes implicated in specific functional roles are very likely to exist even though they have not yet been identified due to limited amounts of sequence homology, a consequence of short pyrosequencing reads. Here MG-RAST was able to assign 9% of sequences to a subsystem and the relative abundances of sequences assigned to each major subsystem were determined (Figure 1). The remaining sequences were identified as hypothetical proteins. The greatest proportion of sequences that could be assigned to a metabolic function were virulence associated (15%). In light of the fact that viruses infect their host cells it was expected that a variety of virulence associated genes would be identified in any viral metagenome. Virulence genes are important both to viral pathogenesis and to host cells acquiring new pathogenic potential following viral infection. However, the rest of the functional families are slightly more surprising on first inspection. The second most abundant function grouping contains genes associated with carbohydrate and protein metabolism (both 10%). Respiration and phosphate metabolism associated genes made up 5 and 3%, respectively, with motility and chemotaxis associated genes representing 2% of the metabolic profiles. Bacteriophages are known modulators of bacterial virulence [52], but they can also carry genes involved in bacterial phosphate metabolism and photosystems [53], both of which were identified in the functional gene analysis of this virome (Figure 1). Analysis of the viral population structure (Figure 2) demonstrates that phages known to carry these genes are also present. *Roseobacter* phage SI01 [NC_002519],** has previously been shown to encode four predicted proteins involved in phosphate metabolism (RP Pho, RP ribonucleotide reductase, RP Thy1 and RP endodeoxyribonuclease 1), suggesting that phosphate recycling is important to the roseophage SI01 life cycle. The published genome sequence of S-PM2 [NC_006820], a bacteriophage that infects marine *Synechococcous* strains, encodes the D1 and D2 proteins of PS11, a large protein pigment complex. The presence of this virus in the host may ensure that photo-inhibition does not occur, enabling photosynthesis to proceed during viral infection, providing the energy needed for virus replication [53].

The presence of motility and chemotaxis genes (17%) (Figure 1) was unexpected as phages and viruses are non-motile, however a functional study of nine viromes by Dinsdale *et al.*, [26] reported a total of 130 SEED annotated motility and chemotaxis proteins present in these metagenomes. The role that they play remains unclear, however, they all carry out functions associated with type 1V pilus and motility, and may be acting as symbiotic relationship facilitators [54]. For example, the pathogenicity of *Vibrio cholerae* is dependent upon the production of a type 1V pilus, and cholera toxin, and the genes for both are encoded on remnant and infective phages, respectively [55]. In general, the assignment of putative functions to a number of the sequences in this virome suggests that there is little restriction on the types of genes carried by a viral community, and their potential for influencing a wide range of biological processes.  

### 2.5. Population Diversity and Structure

In addition to directly causing disease in plants and animals, viruses are important microbial predators that influence global biogeochemical cycling and drive microbial evolution [25]. Much of our knowledge about the roles and diversity of viruses in the natural environment is informed by studies on marine microbial communities. For example 1 kg of marine sediment may contain over a million different viral types, and 200 L of seawater *ca.* 5,000 viral types [28,29], with the vast majority (> 75 %) of genetic material remaining completely uncharacterized. Other environments, including those more directly impacted by human activity, possess greater viral diversity and novelty [56].

**Figure 3:**
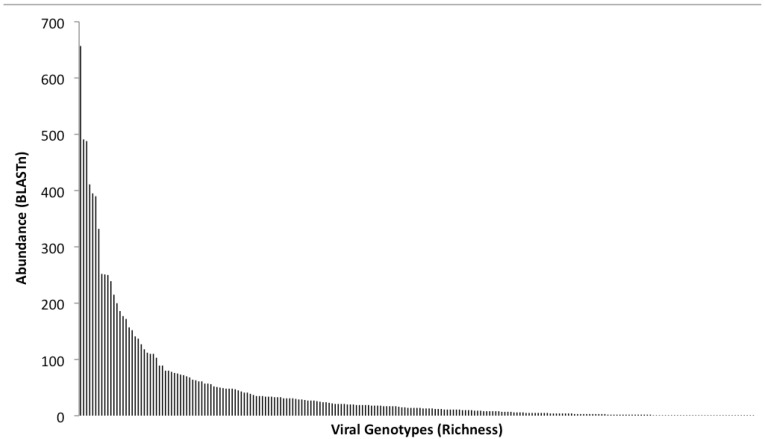
Rank abundance analysis of BLASTn output. This plot was generated from identifications made by comparison of the 454-sequence dataset against the non-redundant viral genome database. Each bar along the X-axis represents a particular viral genotype and the Y-axis indicates the number of representative sequences identified.

The original BLASTn output, which was generated from the comparison of the dataset against the non-redundant viral genome database, was used to perform a rank abundance analysis of family hits (Figure 3). Rank abundance analysis provides a measure of the total number of organisms (abundance = 42,415 viral genotypes), the number of species within the sample (richness = 785 viral genotypes), and a measure of the relative proportional abundances of the species (evenness) [56]. The T4 like phages dominated the metagenome, as did the cyanophages with 411 and 408 representatives in the virome, respectively. Using regression analyses, and assuming the maximal Y value is calculated at X = 1 and the maximal X value is calculated when Y = 1, the formula y = 5,079.7 (X^-1.197^) was used to determine from the abundance plot (Figure 3), an estimate of the number of sequences that would have been obtained if 100% of the population had been sampled. This analysis implied that the true diversity of species present was grossly underestimated, as at least 820,000 sequences would be required to ensure recovery of a single sequence fragment from each member of the viral community. A qPCR-based methodology was previously used to quantify the number of lambdoid bacteriophages in the same viral DNA preparation [11] used to generate the virome, thus enabling an independent assessment of the depth of coverage achieved by the 454 pyrosequencing run. BLAST comparisons of the virome dataset revealed 20 lambda- like sequences and 1 Stx phage, and by comparing this with the absolute numbers of lambdoid and Stx phages determined by qPCR [11], it is estimated that the depth of the virome sequencing has resulted in *ca.* 5% of the viral population in the 2 L of pond water has been sampled. This compares favorably with the theoretical estimate of coverage obtained by the rank abundance analysis (Figure 3) of *ca.* 8%. 

**Figure 4:**
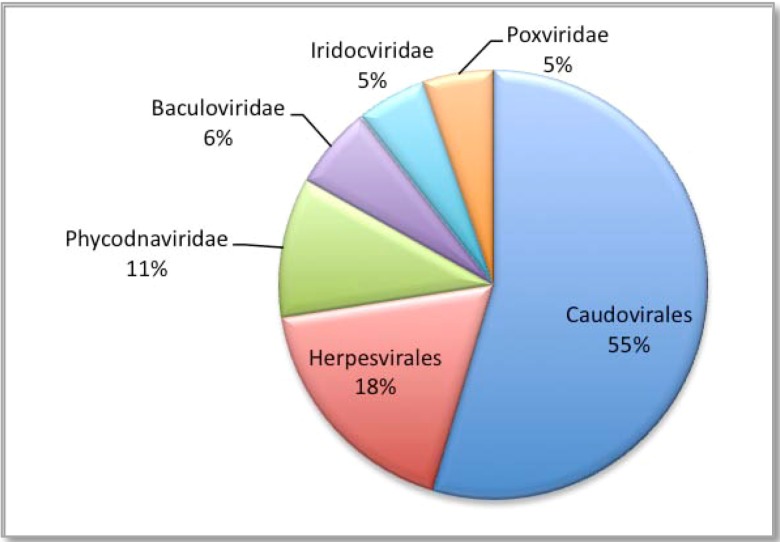
Percentage of sequences assigned to the dsDNA viruses.

All sequences (69,162), were compared to the non-redundant viral genome databases, using a BLASTn algorithm, and in accordance with MEGAN recommendations [39], relaxed alignment parameters were used for the BLAST search. Of the 69,162 sequences generated from the 454 output, 42,715 sequences provided hits from the BLASTn search. These results were then loaded into MEGAN and the lowest common ancestor****(LCA) algorithm assigned 20,287 sequences to taxa with 22,428 remaining unassigned. The LCA algorithm has an inbuilt threshold that is sufficiently stringent to reduce the chances of generating false identities, hence the assignment of the sequences to taxa is both reliable and conservative. Of the assigned sequence reads, 19,010 (93.7%) were identified as dsDNA viruses (Figure 4), of which 10,525 (55.3%) belonged to the *Caudovirales*; 3,459 (18.2%) *Herpesvirales*; 2,034 (10.7%) *Phycodnaviridae*; 1,197 (6.3%)* Baculoviridae*; 1,026 (5.4%) *Iridoviridae* and 1,028 (5.4%)* Poxviridae.*

The primary viral families in this virome associated with infection of metazoans where the *Herpesviridae,* a large family of DNA viruses that cause disease in animals and humans worldwide [57]. In this sample of pond water (accessed by cattle for drinking water), this viral family was represented in 18.2% of the classified virome. All four previously described bovine herpes viruses (BHV-1; BHV-2, BHV-4 and BHV-5) were identified (Figure 2) and are known to cause disease in cattle, worldwide [58]. Of these, bovine herpesviruses 1 (BHV-1 [NC_001847]) and 5 (BHV-5 [NC_005261]) are two closely related viruses that infect cattle [59] and cause multiple diseases including infectious bovine rhinotracheitis, abortion, infectious vulvovaginitis and systemic infection in bovine neonates [60]. When animals survive a BHV infection, a life-long latent infection is established in nervous sensory ganglia, providing both a viral reservoir and survival strategy [60]. Viral reactivation of BHV can occur at any time, and the virus can be shed and transmitted to both immune and non-immune hosts [61]. The *Phycodnaviridae*, are a rapidly expanding collection of large icosahedral dsDNA viruses which infect algae [62] and here comprised 10.7% of the classified virome, mostly represented by different species of the *Chloroviruses*, ubiquitous in nature and isolated from freshwater throughout the world [62]. 5.4% of the classified virome population was comprised of members of the *Iridoviridae and* all were members of the group *Ranavirus*, pathogens which have affected amphibian populations worldwide [63]. Also present in the population are members of the *Poxviridae* (5.4%), a group of established pathogens that includes zoonotic forms that infect animals and humans [64]. *Baculovirdae,* a family of large rod shaped viruses, comprised 6.3% of the classified virome; these viruses are extremely species specific, with larval forms of *Lepidoptera* (moths) the most common hosts [65]. These data suggest that the water in the cattle pond can serve as a reservoir for viruses that directly affect metozoans.

DNA sequences identified with the tailed bacteriophages (Caudovirales), predominate (93.17%) in the cattle pond virome (Figure 2), and comprise the *Myoviridae*, *Siphoviridae* and *Podoviridae*-like viruses. The *Myoviridae* were the most represented** (53%), predominantly T4-like viruses (58% of the *Myoviridae* sequences) with exemplars such as *Synechococcus* phage S-PM2 and *Prochlorococcus* phages P-SSML. Additional members of the *Myoviridae* whose presence was indicated, included the Spo1-like viruses that infect *Bacillus spp.* The *Siphoviridae*, characterized as having long non-contractile tails, made up 30% of the identified *Caudovirales,* dominated by the mycobacteria-like phages (72%). Members of the *Siphoviridae* which infect mycobacterial hosts have proven useful for the diagnosis of mycobacterial infections, including bovine tuberculosis, a zoonosis, which can spread to humans through inhalation of infectious droplet nuclei and by ingestion of raw milk [66]. To date, 50 mycobacteriophages have been fully sequenced, and sequences matching 11 of these are represented in the virome (Figure 2) [67,68]. Analysis of the sequenced genomes suggests that these viruses may also play a more important role in human disease than originally thought. CJW1 [NC_004681] and phage omega [NC_004688] (Figure 2) encode close homologs to the leprosy and tuberculosis antigen Lsr2 suggesting a role for phage in Mycobacterial virulence, and phage Bxz2 [NC_004682] (Figure 2) encodes a homolog with ~35% identify to the human Ro protein, which has major involvement in the autoimmune response in Lupus and Sjögrens syndrome [69]. Whether these genes are involved in virulence is unknown at present, but there is precedence for phage encoded genes being important in diseases, for example, the ability to produce Shiga toxin (Stx), ultimately the most important virulence factor of enterohaemorrhagic *E. coli*, is conferred to *E. coli* following infection with Stx-bacteriophage(s), lambdoid phages that carry the Shiga toxin operon [52]. These are a heterogeneous group of phages with *stx* gene carriage as their common property [11,52], but there is evidence that short-tailed phages (*Podoviridae*) are the most epidemiologically significant [35,70]. The virome contained sequences originating from lambda-like phages (20) and a single *stx* gene sequence, and as discussed above, estimates of the population of lambdoid and Stx phages obtained by qPCR analysis in this pond water sample [11] were used to determine the depth of sampling achieved in the 454 pyrosequenced virome. The *Podoviridae* sequences in the virome were dominated by the *Roseobacter* S101-like, and *Thalassomonas* BA3-like phages [NC_009990] (Figure 2), perhaps indicating that even in water directly contaminated by cattle faeces (thereby contaminated with bovine-associated gut bacteria and bacteriophages), the bacteriophages infecting indigenous freshwater bacterial species predominate the viral community. Nevertheless, these data indicate that the cattle pond can serve as a reservoir for viruses with an indirect pathogenic phenotype for higher life-forms, e.g. temperate phages that directly alter the pathogenic phenotype of their bacterial hosts. This mechanism, coupled with the ability the cattle farm pond to harbor viruses infecting metazoan life, demonstrates the potential of water sources to serve as reservoirs for viruses, and sites for dissemination to new hosts, directly (via viral pathogens) or indirectly (via temperate phages). 

## 3. Experimental Section 

### 3.1. Viral DNA Extraction

Two litre water samples were collected from a farm in Cheshire, U.K. in August 2008 [9]. NaCl_2 _(0.5M) was added to each sample and allowed to dissolve to facilitate the dissociation of viral particles from cellular and particulate surfaces [11,71]. Cell and environmental debris were removed by slow speed centrifugation (6000 x g for 5 min). DNase (Ambion) and RNase (Sigma) were added to a final concentration of 5 µg mL^-1^, and the samples were incubated at 37°C for 30 min. Polyethylene glycol (PEG) with an average molecular weight of 8000 (Sigma) was gradually added to a final concentration of 10% (w/v), and the sample was incubated for 18 h at 4 °C. The sample was centrifuged (10,000 x g for 10 min at 4°C), the supernatants discarded and the pellets suspended in 7.5 mL TBT buffer (100 mM Tris-HCL, pH 7.0; 100 mM NaCl_2_; 100 mM MgCl_2_). An equal volume of chloroform was added, the samples were subjected to centrifugation (4000 x g, 20 min), and the top phase was harvested [11]. A second DNase (Ambion) and RNase (Sigma) step was carried out as described above. Viruses were precipitated by incubation with 33% (w/v) PEG on ice for 60 min and harvested by centrifugation (10,000 x g; 10 min at 4°C). A third DNase and RNase digestion was performed, followed by extraction using an equal volume of equilibrated phenol:chloroform:isoamyl alcohol (25:24:1, pH8.0). The mixture was centrifuged (13,000 x g for 5 min), and the aqueous phase harvested and subjected to a further three rounds of phenol:chloroform:isoamyl alcohol extraction. Viral DNA was precipitated by the addition of equal volumes isopropanol, 10% (v/v) 3 M sodium acetate (pH 5.2.) and glycogen (20 mg mL^-1^) followed by incubation at -20°C for 18 h. The sample was centrifuged (10,000 x g for 30 min at 4°C). The resultant viral DNA pellet was washed with ice-cold 70% ethanol, air-dried and suspended in 15 µL of sterile dH_2_0. 

### 3.2. End-Point polymerase chain reaction

To demonstrate the removal of contaminating prokaryotic or eukaryotic genomic DNA from the viral DNA sample, PCR amplification was performed using universal primer sets specific to bacterial 16S and eukaryotic 18S rRNA genes (Table 1) and Phusion high fidelity DNA polymerase (Finzymes). *Bacillus subtilis* genomic DNA, and DNA extracted from bovine rumen fluid were used as controls for the 16S and 18S amplifications respectively. Each reaction (50 µL) contained forward and reverse primers (0.5 µM, each), dNTP’s (200 µM), HF buffer (Finzymes), and 0.02 U µL^-1^ Phusion high fidelity polymerase. Cycling conditions were 98°C for 30 s, 35 cycles (98°C for 5 s, 1 min at the specific annealing temp for each primer (Table 1), 72°C for 1.5 min) and a final extension step of 72°C for 10 min. Amplification products were detected on a 1% TAE agarose gel (40 mM Tris base, 20 mM glacial acetic acid, 1 mM EDTA, 1.5% (w/v) agarose) run for 1 h at 100V.

### 3.3. pyrosequencing 

Sequencing was performed using the Roche 454 GS-FLX instrument at the NERC funded Advanced Genomics Facility at the University of Liverpool, according to the method for pyrosequencing by Marguilies *et al.* [72].

### 3.4. Bioinformatical analyses 

A BLAST comparison was performed using the BLASTn algorithm [41], against the non-redundant viral genome database [42]. The output of this analysis was used for two different analyses. First, the rank abundance data was generated using the top 15 hits (e< 0.1) of each sequence fragment to identify viral genotypes. Secondly, the output file was uploaded into a windows version of the MEGAN program [39], which was used to estimate and interactively explore the taxonomical distribution of the dataset. The program uses a simple algorithm that assigns each read to the lowest common ancestor (LCA) of the set of taxa with matches in the comparison. The LCA parameters used were: min support, 5; min score, 35.0; top percent, 10.0; win score, 0.0. 

### 3.5. MG-RAST 

All freshwater DNA 454 sequences were uploaded in a FASTA format to the MG-RAST server at the SEED [43].****The raw data presented here can be found on the MG-RAST (Public: MGPhage (4442702.3) from project PhageMG).

## 4. Conclusions 

This paper provides a snapshot examination of the viral diversity present in a single sample of DNA harvested from a freshwater pond on a cattle farm in Cheshire, UK. A broad range of viruses were found that are directly and indirectly associated with diseases affecting plants, animals and man, including Stx phages, *Herpesviridae*, and *Poxviridae.* These data support the concept that the aquatic environment plays a more important role as a reservoir of viral pathogens than may have been previously been appreciated. In addition to directly causing disease, viruses are also important predators, controlling microbial population size and structure, influencing global biogeochemical cycling and driving microbial evolution through gene transfer. Analysis of functional potential of genetic information within the virome identified homology to genes associated with virulence, photosystems, metabolism and even motility. Freshwater viral diversity and community structure has barely been studied and even though this virome possessed a high diversity of viral genotypes (785), rank-abundance analyses indicated that the depth of sequencing employed enabled only *ca.* 8% of the virus population in the 2 L freshwater sample to be examined. Less than 62% of the 69,162 sequences (42,715 sequence reads) were assigned a BLASTn hit using the viral databases. Less than 30% of 69,162 reads could be assigned to a function/genotype via SEED (18,931 sequence reads) and MEGAN (20,287 sequence reads). Techniques like those reported here, represent a fundamental breakthrough in our ability to detect and monitor viral populations. Further work on determining abundances linked with disease monitoring and viral dynamics may be integral to the development of risk analysis tools. Delivery of this information to the end users, such as government advisory bodies for environment, human health, water safety, control of recreational water and agriculture will enable us to pre-emptively deal with disease affecting and impacting upon animals, plants and humankind.

## Supplementary Files

Supplementary File 1 Figure 2 (PNG, 975 KB)

Supplementary File 2 Supplementary Table 1 (XLSX, 5179 KB)

Supplementary File 3 Supplementary Table 2 (XLSX, 360 KB)
