# Supplementary figures and images for: 454-Pyrosequencing: A Molecular Battiscope for Freshwater Viral Ecology
Source: Genes (Basel). 2010 Jul 21;1(2):210–26. doi: 10.3390/genes1020210 (PMC3954088; doi:10.3390/genes1020210)

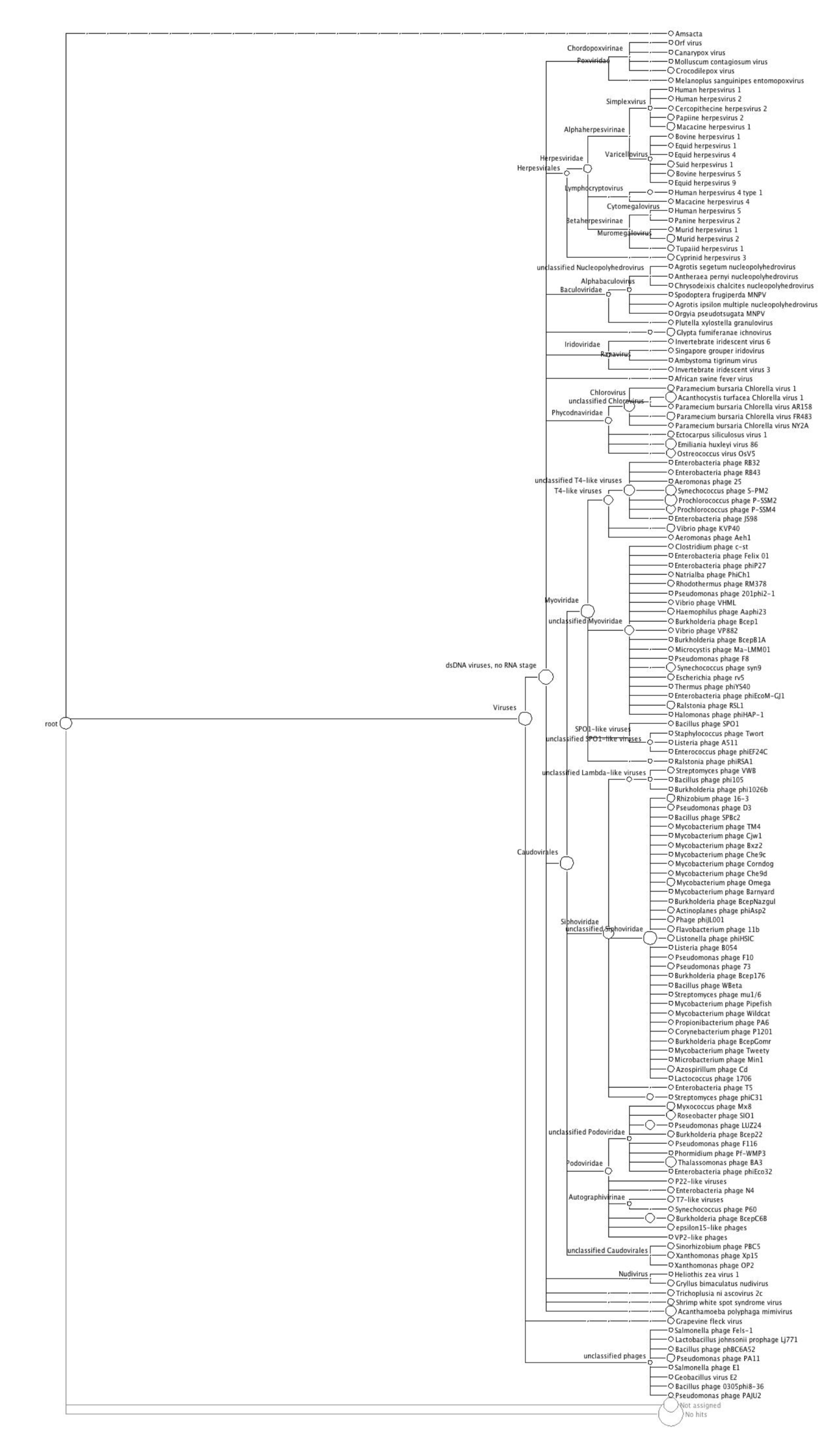

Supplement: Supplementary File 1 — Figure 2 (PNG, 975 KB) [file genes-01-00210-s001.png]
